# 

**Published:** 1897-08

**Authors:** 


### CONTENTS.

ORIGINAL ARTICLES:

The Nerve Element in Surgical Pathology........................ 371

The Crepitant or Vesicular Rale an Inter-pleural Sound and not Caused in the Pulmonary Tissue.................................. 373

SOCIETY REPORTS:

The Atlanta Society of Medicine ........................... 379

Regular Meeting of the Atlanta Society of Medicine............. 383

SELECTIONS AND ABSTRACTS:

Sub-Peritoneal Myoma.......... ....... .. .................... 389

The Glandular Fever of Childhood.................. ............ 392

Woman's Place in Medicine.................. ... ................ 394

Chronic Malarial Toxemia, its Prevalence in New York City—Causes and Treatment by Alteratives.................... .. 396

Some Water Uses Well to Remember............................. 397

Infantile Diarrhœa.............................................. 398

The Question of Treating the Poor Who Can Not Pay the Physician...................................................... 398

Castration for Rape in Kansas.................................. 399

Operative Treatment of Facial Neuralgia.............. ........ 400

Liability for Maliciously Filing Information Charging Insanity.. 401

Lime-water as a Disinfectant for Linen and Cotton Fabrics..... 401

Fashions and Fancies in Food........... ......................... 402

Summer Complaints—Prophylaxis................................ 403

Competition for the Senn Medal... ........................... 405

EDITORIAL:

Death of Father Kneipp...................... ................. 406

Euthanasia............... ........................................ 408

BOOK REVIEWS.................................................. 411

BUSINESS DEPARTMENT................................ ........ 416

PRESCRIPTION DEPARTMENT................................. 421

